# A new technique to prevent conjunctival prolapse in Asian patients for correcting severe blepharoptosis

**DOI:** 10.1186/s12886-024-03318-8

**Published:** 2024-02-05

**Authors:** Wenwen Xi, Ziqing He, Feng Yang

**Affiliations:** Department of plastic and cosmetic surgery, The Second Affiliated Hospital of University of South China, Hengyang, Hunan 421000 China

**Keywords:** Blepharoptosis, Conjoint Fascial sheath( CFS) Suspension, Conjunctival Prolapse, Complication

## Abstract

**Background:**

In Asian patients with severe ptosis,the use of conjoint fascia sheath (CFS) suspension or levator aponeurosis fascia complex shortening surgery can correct the ptosis. During these surgery, a significant amount of levator aponeurosis fascia shortening is performed, which often leads to serious complications such as conjunctival prolapse.This study compares two surgical approaches for correcting severe blepharoptosis:Conjoint fascial sheath (CFS) + levator aponeurosis and muller’s muscle complex (LM complex) suspension and conjoint fascial sheath (CFS) + LM complex+conjunctival suspension.The postoperative efficacy and the incidence of complications such as conjunctival prolapse are investigated for both procedures.

**Methods:**

This study retrospectively analyzed 70 patients (77eyes) with severe blepharoptosis from January 2019 to December 2021. The patients were divided into the experimental group (34 cases, 38 eyes) and the control group (36 cases, 39 eyes). The experimental group was treated with CFS+LM complex + conjunctival suspension, and the control group was treated with CFS+LM complex suspension.The curative effect of blepharoptosis, the incidence of complications such as conjunctival prolapse and patient satisfaction were compared between the two different surgical methods.

**Results:**

There was no significant difference in the correction effective rate between the experimental group (84.21%) and the control group (82.05%) (*P >* 0.05). There was no significant difference in the total incidence of complications between the experimental group (23.68%) and the control group (38.46%) (*P >* 0.05), but in the complication of conjunctival prolapse, the incidence of conjunctival prolapse in the experimental group was significantly lower than that in the control group. The difference was statistically significant (*P <* 0.05). In the survey of patient satisfaction rate, the satisfaction rate of the experimental group was significantly higher than that of the control group,which was statistically significant (*P <* 0.05).

**Conclusions:**

Compared to CFS+LM complex suspension surgery, the CFS+LM complex + conjunctival suspension has a definite effect in preventing postoperative conjunctival prolapse .The procedure has a high feasibility, good corrective effect, and improves patient satisfaction after surgery.

**Supplementary Information:**

The online version contains supplementary material available at 10.1186/s12886-024-03318-8.

## Introduction

Conjunctival prolapse can occur after corrective surgery for severe blepharoptosis. During the surgery, methods such as shortening the levator aponeurosis complex or suspending CFS+LM complex are commonly used to correct severe blepharoptosis. However, during the surgical process, the surgeon often shortens the levator aponeurosis complex significantly without addressing the length of the conjunctiva in that area. As a result, long conjunctiva may prolapse after surgery [1–3]. In this article, we described our experience of CFS+LM complex+conjunctival suspension in the treatment of severe blepharoptosis, and evaluated the postoperative outcomes and the incidence of complications such as conjunctival prolapse compared with the CFS+LM complex suspension surgery, so as to guide how to prevent conjunctival prolapse in severe blepharoptosis correction in clinical work.

## Patients and methods

### Materials

A total of 70 patients (77 eyes) with severe blepharoptosis in the Medical Beauty Center of our hospital from January 2019 to December 2021 were collected. the patients with bilateral or unilateral severe blepharoptosis were selected. According to the method of operation, the patients were divided into two groups: the experimental group (34 cases, 38 eyes) and the control group (36 cases, 39 eyes). There was no significant difference in general data between the two groups (*P >* 0.05) (Table 1).

**Table 1:** Comparison of general clinical data between the two groups [cases (%)]

Group	Number of cases	Average age (years)	Number of affected eyes (eyes)	Gender		Types of ptosis	
				male	female	Unilateral severe ptosis	Bilateral severe ptosis
The experience group	34	25.744.97	38	12(35.29)	22(64.71)	30 (88.24)	4 (11.76)
The control group	36	24.974.97	39	11(30.56)	25(69.44)	33 (91.67)	3 (8.33)
P		0.523		0.673		0.632	

### Diagnostic criteria

MRD1 (Margin Reflex Distance1,MRD1),MRD1 is the distance from the light reflex on the patient's cornea to the central portion of the upper eyelid margin with the patient gazing in the primary position. Normal MRD1 is 4–5 mm [4]. Those with MRD1 of 1 mm or less were defined to have severe ptosis [5, 6].

### Exclusion criteria

The patient had no prior history of blepharoptosis surgery, and no history of eyelid trauma, facial nerve dysfunction, mandibula-blink syndrome, blepharospasm, Horner syndrome or myasthenia gravis. All operations were performed by the same experienced surgeon, and all patients signed informed consent.

### Preoperative assessment and design

According to the MRD1 and the strength of the levator palpebral muscle of the patient, The amount of levator aponeurosis shortening needs to be evaluated. In patients with bilateral severe blepharoptosis, the double eyelid width is designed to be 4~5mm,the width of the double eyelid line is designed to be 4-5 mm, and the heavier the degree of ptosis, the narrower the design width of the double eyelid.In unilateral patients, the incision is designed according to the shape of the healthy eyelid or according to the requirements of the patients.

### Surgical technique

1. In the experimental group, CFS+LM complex+Conjunctival suspension were used to correct severe blepharoptosis.

**Fig. 1:**
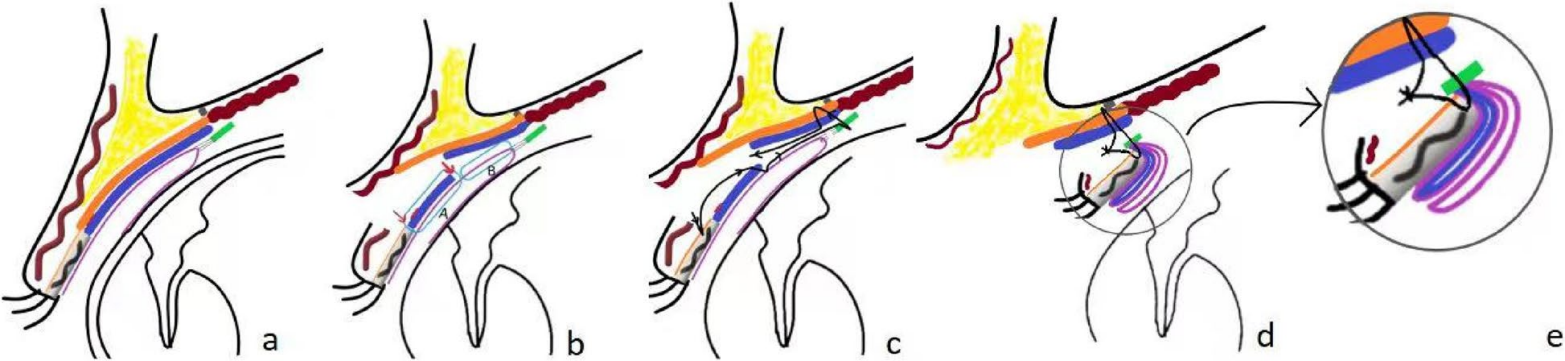
Is a schematic diagram of intraoperative suture technique. **a** is a sagittal view of normal upper eyelid structure, in which orange lines represent levator aponeurosis, blue lines represent muller’s muscle, purple lines represent conjunctiva, and green lines indicate CFS; red short lines represent peripheral arterial arch. **b** is the upper eyelid structure after incision of levator aponeurosis and muller’s muscle. The thin arrow indicates the position of the incision of the levator aponeurosis, and the thick arrow indicates the position of the muller’s muscle from the 8~10mm of the superior edge of the tarsal plate. Part A was mullers muscle and conjunctiva, and part B was only conjunctival tissue. **c** is the position of suture. **d** is the double "Ω" -shaped tissue structure after suture. **e** is the enlarged double "Ω" -shaped schematic diagram

**Fig. 2:**
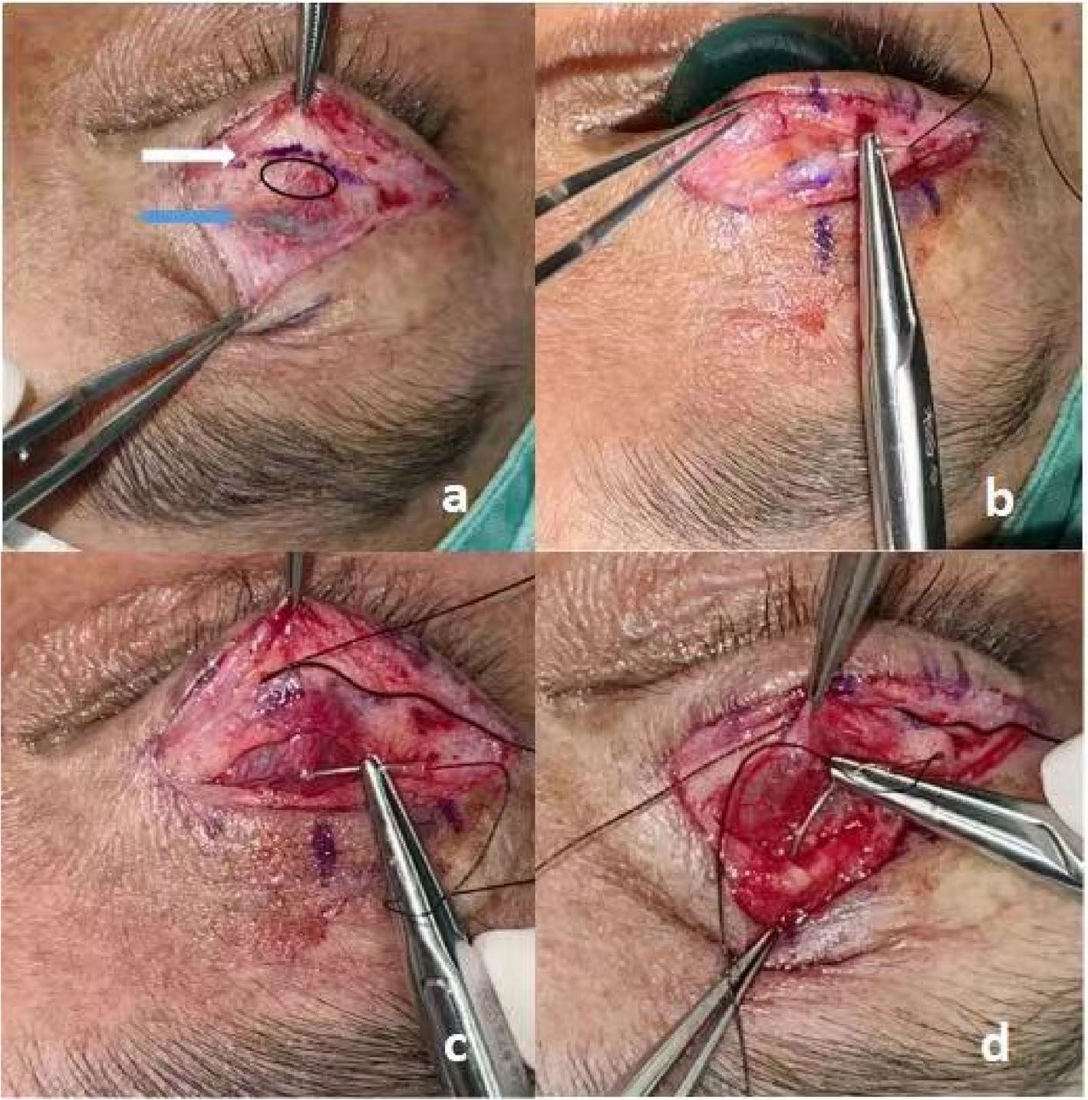
**a** is a picture of the operation, the white arrow indicates the incision of the levator aponeurosis, the blue arrow indicates the incision of the muller’s muscle at the 10mm above the superior edge of the tarsal plate, and the black ellipse represents the peripheral arterial arch. **b** is in the position of suturing the upper part of the tarsal plate; **c** is in the suture of muller’s muscle and conjunctival complex; **d** is in the suture of CFS and LM complex

Fifteen minutes before surgery, obucaine eye drops were dropped into the eyeball for conjunctival surface anesthesia.The incision subcutaneous and pretasal infiltration anesthesia was performed by injecting the 2% lidocaine with 1:100,000 adrenaline.The skin was incised, excess orbicularis oculi were excised. and then,the superior edge of the tarsal plate was exposed. (Fig. 2a white arrow) The orbital septum was completely opened upward, the levator aponeurosis was cut at the superior edge of the tarsal plate. (Fig. 1b thin arrow) Then,the upper eyelid was turned over, and the 2% lidocaine was injected under the conjunctiva,which is also called ”the water separation”.The muller’s muscle was incised 8~10mm above the superior margin of the tarsus. (Fig. 1b thick arrow; Fig. 2a blue arrow) The structure between the muller’s muscle and conjunctiva was loose after the water separation. We separated into the fornix along the space between the conjunctiva and muller’s muscle until the white thickened fibrous connective tissue(CFS) was exposed.The upper 1/3 of the tarsal plate,muller’s muscle and conjunctival complex, CFS, levator aponeurosis and muller’s muscle complex (LM complex)are sutured together in the middle position of the upper eyelid using 5-0 silk thread (Fig. 2b-d).If the position of the middle fixation suture was appropriate, additional 4 fixation sutures were added on each side of the first fixation suture.In this way, the upper 1/3 of the tarsal plate, Müller's muscle and conjunctival complex, CFS and LM complex form a double "Ω" -shaped parallel double-loop structure,constituting a double-layered folded suspensory conjunctiva. (Fig. 1a-e). The double eyelid was made with 6 stitches of 7-0 nylon between orbicularis oculi muscleand the pretarsal fascial tissue, and then reinforced with the final skin-orbicularis oculi muscle-pretarsal fascia-orbicularis oculi muscle-skin closure with interrupted 7-0 protein sutures.

2) In the control group, CFS+LM complex suspension was used to correct severe blepharoptosis.

After anesthesia in the upper eyelid surgical area, the skin and orbicularis oculi muscle are carefully incised,The LM complex is then cut at the superior edge of the tarsal plate and separate into the conjunctival fornix until the CFS is exposed. According to the height of eyelid correction, the upper 1/3 of the tarsal plate, CFS and LM complex are sutured with 5-0 silk thread. Unlike the experimental group, no conjunctival tissue is included here. Finally, the incision is sutured layer by layer with 7-0 nylon thread and 7-0 prolene suture.

In addition, during the first week after surgery, a 3-0 silk suture is placed in the lower eyelid to close the palpebral fissure in both the experimental group and the control group After surgery, eye drops and ointment should be used until the eyelids are completely closed or until no signs of pupil are visible.

### Follow-up

Two senior plastic surgeons jointly evaluated the correction of upper eyelid ptosis and the occurrence rate of complications such as conjunctival prolapse in two groups of patients. Patient satisfaction was assessed through self-evaluation scores, with a follow-up period of 3 to 12 months. Evaluation criteria for the correction of ptosis: Good correction: the upper eyelid of both eyes are highly symmetrical, the curve is smooth and natural, the upper eyelid covers the upper margin of cornea 1-2mm.General correction: the height of the upper eyelid is basically symmetrical, the radian is natural, the difference between MRD1 and normal value is less than 2mm, and the degree of ptosis is better than that before operation. Insufficient correction: the upper eyelid margin was covered to the upper margin of the pupil, and the difference between MRD1 and normal value was more than 2mm. Over correction: the upper eyelid margin was more than 1 mm above the corneal limbus [4]. Good correction and general correction are effective correction, correction efficiency = (good correction + general correction) / total number of cases × 100%.

In the evaluation of patient satisfaction, the subjective improvement degree of patients was > 70%, 50%~70% was general, and the patient satisfaction rate was = (satisfaction + general) / total number of cases × 100%.

### Statistical method

The data of this study were analyzed by SPSS 26.0 statistical software. the measurement data were expressed by (X ±s), the counting data were expressed by T test, the counting data were expressed by (%), and the difference was statistically significant by χ 2 test.

## Results

**Fig. 3:**
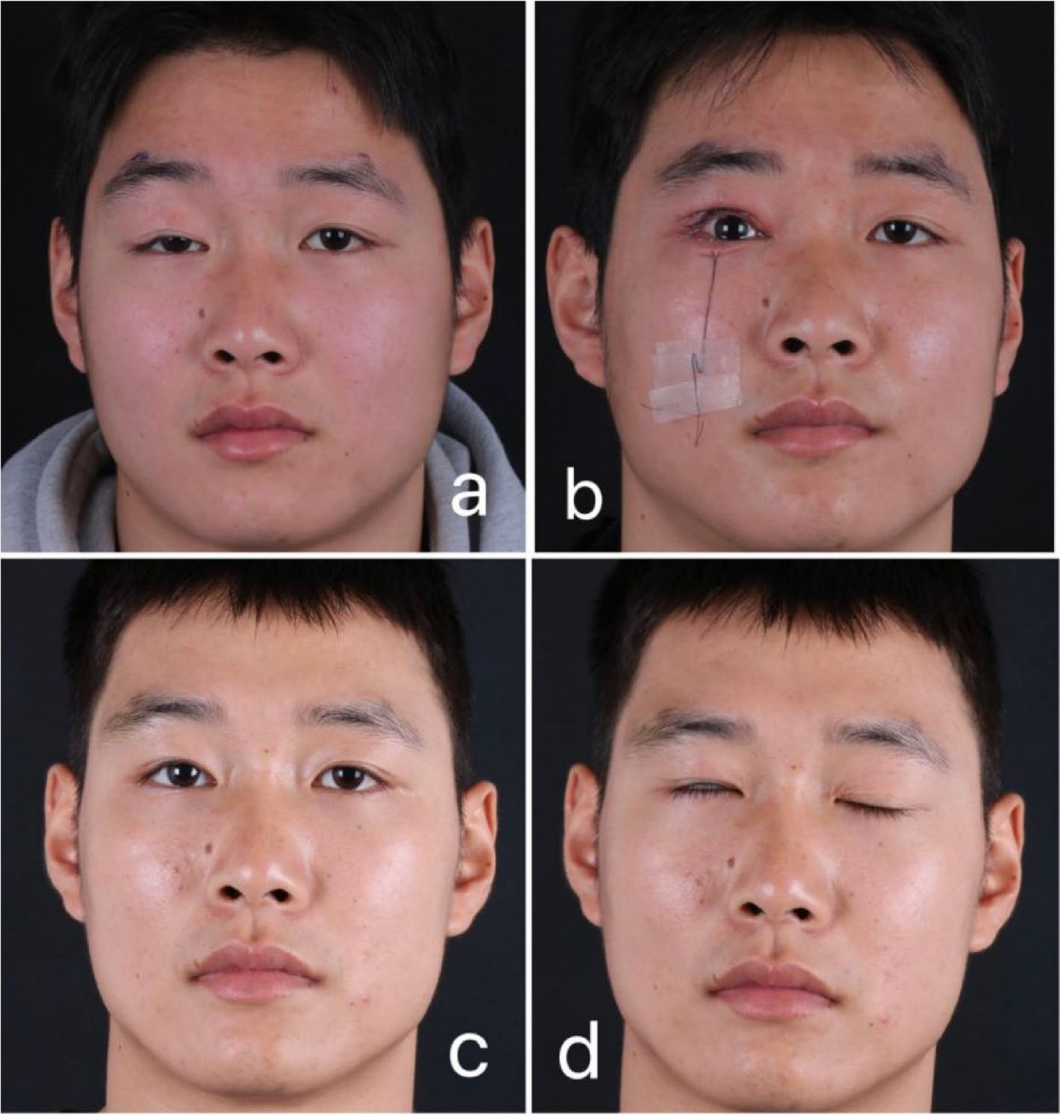
Shows a 25-year-old male with severe ptosis on the right. **a** is taken before operation; **b** is taken immediately after operation; **c** is eyes opened 6 months after operation; **d** is closed 6 months after operation

A total of 77 eyes of 70 patients in our center underwent severe ptosis correction using CFS+LM complex + conjunctival suspension in the experimental group, and CFS+LM complex suspension in the control group. The patients were followed up for a duration of3 ~ 12 months (Fig. 3a-d). The results showed that there was no significant difference between the experimental group (84.21%) and the control group (82.05%) (*P >* 0.05) (Table 2). In both groups, all eyes experienced varying degrees of upper eyelid swelling and bruising within 1 week after the surgery, which gradually disappeared within 1 month. There was no significant difference in the overall incidence of complications between the observation group (23.68%) and the control group (38.46%) (*P >* 0.05). However, in terms of the specific complication of conjunctival prolapse, no cases were observed in the experimental group, while 5 cases of conjunctival prolapse occurred in the control group within 1 week.The incidence of conjunctival prolapse in the experimental group (0.00%) was significantly lower than that in the control group (12.82%) (*P <* 0.05) (Table 3). When evaluating patient satisfaction, the experimental group had a significantly higher satisfaction rate (89.47%) compared to the control group (71.79%), and the difference was statistically significant (*P <* 0.05) (Table 4).

**Table 2:** Comparison of correction of severe ptosis between the two groups [n (%)]

Group	Number of affected eyes n (eyes)	Good Correction	General Correction	Insufficient Correction	Over Correction	Effective rate (%)
The experience group	38	20	12	5	1	32(84.21)
The control group	39	21	11	6	1	32 (82.05)
χ^2^	0.064					
P	0.800

**Table 3:** The incidence of complications was compared between the two groups

Group	Number of affected eyes n (eyes)	Incomplete closure of palpebral fissure	Asymmetry of double eyelid curvature	Conjunctival prolapse	Exposure keratitis	Total complication rate (%)
The experience group	38	2	7	0	0	9(23.68)
The control group	39	3	6	5	1	15(38.46)
χ^2^		0.187	0.126	5.210	0.987	1.959
*P* value		0.665	0.722	0.022	0.320	0.162

**Table 4:** Comparison of patient satisfaction between the two groups [n (%)]

Group	Number of cases	satisfied	general	dissatisfied	Satisfaction rate (%)
The experience group	38	25	9	4	34 (89.47)
The control group	39	21	7	11	28 (71.79)
χ^2^	3.966				
*P* value	0.046				

### Discussion

Ptosis, a common eye disease, can have varying degrees of impact on individuals.Mild ptosis mainly affects one’s appearance, while moderate and severe blepharoptosis can partially or completely obstruct the visual field, significantly impacting the development of vision in patients. Over time, this condition can lead to other issues such as strabismus, amblyopia, head tilting and forehead lifting, and even spinal deformity and scoliosis [6, 7]. According to the literature, the incidence of amblyopia is approximately 2.8% ~ 3.2%.Among patients with ptosis, the incidence of amblyopia ranges from 23.9% to 56%, and the risk increases with the severity of ptosis [7–9].

At present, there are many surgical approaches available for the treatment of blepharoptosis, These include the upper eyelid skin resection as well as more commonly used improved techniques such as frontalis muscle flap suspension, levator aponeurosis advancement, folding and shortening.By use of the muller’s muscle, tarsal resection, CFS and other surgical methods to correct ptosis, each of the above surgical methods has its own indications , advantages and disadvantages [10–14]. In view of the severe ptosis discussed in this paper (The difference of MRD1 between the affected eye and normal eye was > 4mm) [4–6], we used CFS+LM complex suspension operation to correct ptosis.

Currently, the use of levator palpebrae superioris muscle for ptosis correction is the most consistent with the physiological structure of the eye. This method is relatively simple, provides natural postoperative results,and has fewer complications [15]. However, in cases of severe ptosis where the functionality of the levator palpebrae superioris muscle is greatly impaired or non-existent, the correction effect of levator muscle alone can not be achieved. In such scenarios, the CFS suspension operation should be chosen as the preferred method for ptosis correction.The conjoint fascial sheath (CFS) was located in the intermuscular space between the anterior third of the superior rectus and the levator .Posteriorly, it extended from the fascia of the levator and superior rectus. Anteriorly, superficial and deep extensions of CFS continued approximately 2 mm to the superiorconjunctival fornix and then 2–3 mm distally along and beneath the palpebral and bulbar conjunctiva [16]. It was first applied to correct ptosis in 2002, and since then, this technique of CFS suspension has become increasingly popular for severe blepharoptosis [17, 18]. The CFS+LM complex suspension operation uses not only the dynamic strengthening principle of levator muscle shortening, but also the static suspension of tough CFS. This surgical technique ensures a coordinated connection between the initial static suspension height and the long-term muscle strength height, resulting in a relatively consistent upper eyelid margin height. Moreover, the suspension direction aligns with the movement direction of the levator muscle [15–19], which is in accordance with the physiological characteristics of the human body.This approach has the advantage of minimal postoperative complications.

Firstly, what is the relationship between the correction of severe ptosis and conjunctival prolapse with CFS+LM suspension procedure? The study discovered that regardless of the chosen surgical intervention to correct ptosis, it aims to shorten and modify the tissue structure above the conjunctiva, without altering the length of the conjunctival itself.The normal width of the tarsal plate is 8~9mm, whereas the length of the Whitnall ligament from the upper edge of the tarsal plate is approximately 20~22mm [20, 21]. In patients with mild blepharoptosis, only the levator muscle is correctly advanced, folded or shortened, and the fornix conjunctiva overlaps only minimally, typically not extending beyond the upper eyelid margin. In severe blepharoptosis cases, we often advance 15~20mm or even utilize CFS to shorten the levator aponeurosis complex. At this time, the length of the folded conjunctiva in the fornix exceeds the width of the tarsal plate itself. The upper eyelid plate can not fully cover the entire conjunctiva, especially as the lateral side of the tarsal plate is narrower than the middle.This makes the conjunctiva more prone to prolapse,leading to a more notable prolapse of the upper eyelid’s lateral edge post-operation.This constitutes the anatomical basis for conjunctival prolapse in patients with severe ptosis after correction.

Secondly, under normal circumstances, a small fiber connection exists between the CFS and the superior fornix conjunctiva, creating a fornix suspension ligament that maintains the position and stability of the fornix conjunctiva. During severe blepharoptosis correction, the levator aponeurosis, Muller’s muscle and conjunctiva are carefully separated layer by layer, exposing the CFS .In this procedure, the fibrous ligament connecting to the conjunctiva is severed and disrupted, resulting in the loss of upward suspension support for the fornix conjunctiva. Additionally,a potential gap is formed between the levator aponeurosis and the conjunctiva due to the difference in lenth between the shortened levator aponeurosis and the excess conjunctiva [2, 3]. As a consequence of gravity, accumulated tissue fluid and blood graduallycollect in the space, leading to conjunctival prolapse.

Thirdly, the extensive dissection of the levator aponeurosis, muller’s muscle and conjunctiva during the operation,along with the removal of the fornix suspension ligament, increase the release of vascular inflammatory mediators.This, in turn, leads to changes in vascular permeability,a decrease in osmotic pressure, and obstruction of lymphatic reflux function [22, 23]. Consequently, there is continuous fluid exudation, edema, and vasodilation in the subconjunctival space. When the conjunctiva prolapse, it becomes trapped between the upper eyelid and the eyeball, hindering lymph and tissue fluid reflux.This further exacerbates conjunctiva edema, vascular dilatation, and swelling of the prolapsed conjunctiva [24, 23, 25], creating a vicious cycle that worsens conjunctival prolapse.

Based on the author's extensive clinical experience spanning over 20 years, it has been determined that prevention is the key to addressing the issue of conjunctival prolapse following severe ptosis correction. In this study, 70 patients (77 eyes) with severe ptosis were divided into an experimental group and a control group based on the different surgical methods employed. The experimental group underwent treatment with CFS+LM complex+ conjunctival suspension,while the control group received with CFS+LM complex suspension. The following key measures were implemented to prevent conjunctival prolapse during the operation in the experimental group:

1. the integrity of muller’s muscle and conjunctival complex within the 8-10mm range of the superior margin of tarsal plate was preserved for three reasons:
  1. The conjunctiva closely adheres to the muller’s muscle within the 3-5mm range of the superior margin of the tarsal plate.Forceful separation between muller’s muscle and the conjunctival can easily result in conjunctival perforation, compromising its integrity and increasing the risk of postoperative conjunctival prolapse.
  2. There is a parallel peripheral arterial arch in the 3-5mm range of the superior margin of the tarsal plate (Fig. 2a Black circle). This arch is prone to injury when the levator aponeurosis is cut and separated upward, and any bleeding can easily spread upward within the loose space between the levator aponeurosis and muller’s muscle,thereby affecting subsequent procedures.
  3. The complex, with a certain length, becomes thicker, increasing the difficulty of prolapse compared to the thin conjunctiva.
2. Double "Ω" sutures were utilized. During the stitching process. a 5-0 silk thread was inserted into the upper 1/3 of the tarsal plate,passing through the muller’s muscle and conjunctiva complex at the boundary between points A and B (Fig. 1b),the CFS, and the LM complex (Fig. 1c). At this point, the complex tissue between the superior edge of the tarsal plate and the superior fornix was divided into two segments of approximately equal length but varying thickness.Consequently,the conjunctiva was folded in a double layer ((Fig. 1d). The length of the conjunctiva folded downward is only half that of conventional operations, making it difficult to surpass the width of the tarsal plate and result in prolapse.

The reason for preserving the LM complex at a distance of 8-10 mm above the upper edge of the tarsal plate is based on clinical and anatomical studies,which have demonstrated a gradual transition of the levator aponeurosis from a dense, tough and thick white connective tissue to a translucent thin film structure [20]. During the surgical procedure, the levator aponeurosis undergoes a color change from white to red. Forceful separation of the levator aponeurosis from muller’s muscle can easily lead to rupture or even perforation

The results of the 3 to 12 month follow-up in both the experimenta and the control groups indicated no significant difference in the rate of effective correction between the two groups (*P >* 0.05).However, the total incidence of complications in the experimental group was significantly lower than that in the control group (*P <* 0.05),particularly regarding the complication of conjunctival prolapse. The survey on patient satisfaction rates revealed that the experimental group had a significantly higher satisfaction rate compared to the control group (*P <* 0.05). The questionnaire survey further revealed that all patients with postoperative conjunctival prolapse in the control group were dissatisfied, accounting for 45.45% of the unsatisfied patients in the control group.This suggests that postoperative complications of conjunctival prolapse greatly affect patient satisfaction.

**Conclusions**

Preventing conjunctival prolapse during the severe blepharoptosis operation is crucial to avoid this complication.In this study, compared to CFS+LM complex suspension,the addition of conjunctival suspension to CFS+LM complex provides a double "Ω" conjunctival foldingtechnique.This methodhas proven effective in preventing postoperative conjunctival prolapse and other complications.Moreover,the procedure has a high feasibility, good corrective effect, and improves patient satisfaction after surgery.

### Supplementary Information

**Additional file 1.**

## Data Availability

All data generated or analysed during this study are included in this published article.
